# Supplementary material for: Content-rich biological network constructed by mining PubMed abstracts
Source: BMC Bioinformatics. 2004 Oct 8;5:147. doi: 10.1186/1471-2105-5-147 (PMC528731; doi:10.1186/1471-2105-5-147)
Supplement: Additional File 2 — The original results of the above study (non-essential files are deleted to keep the file size under the limit set by BMC bioinformatics). [file 1471-2105-5-147-S2.bz2 › chilibotAdditionalFile2/dip10/591ID9253415E10963/html/TUFA_TSF.html]

 


 **TUFA** and **TSF** 
  
Found 146 abstracts in PubMed, retrieved 10.  
 

 What does Google say? 
 PDF only 
| .edu only 

---

**Interactive relationship** (e.g. stimulation, inhibition, etc)

**Neutral relationship**- Characterization of the interaction between the nucleotide exchange factor EF Ts  [ **TSF** ]  from nematode mitochondria and elongation factor Tu  [ **TUFA** ] .  Ref: 12490713 Nucleic Acids Res, 2002
- The EF Ts  [ **TSF** ]  bound to two eubacterial EF Tu  [ **TUFA** ]  species E.coli and Thermus thermophilus.  Ref: 12490713 Nucleic Acids Res, 2002
- Nucleotide exchange in elongation factor Tu  [ **TUFA** ]  EF Tu  [ **TUFA** ]  is catalyzed by elongation factor Ts  [ **TSF** ]  EF Ts  [ **TSF** ] .  Ref: 11744709 J Biol Chem, 2002
- This indicates that nematode EF Ts  [ **TSF** ]  can be used to stabilize homologous EF Tu  [ **TUFA** ]  molecules for experimental purposes.  Ref: 12490713 Nucleic Acids Res, 2002
- In contrast to earlier experiments, purification of free mitochondrial EF Ts  [ **TSF** ]  was accomplished under nondenaturing conditions since only about 60% of the expressed EF Ts  [ **TSF** ]  copurified with E. coli EF Tu  [ **TUFA** ] .  Ref: 12445479 Biochim Biophys Acta, 2002
- Some of them are in the regions of interdomain interactions, others correlate with the known functionally important regions of EF Tu  [ **TUFA** ]  binding with EF Ts  [ **TSF** ] , aminoacyl tRNA and the ribosome.  Ref: 11771276 Biofizika< MedlineTA>Biofizika, 2002
- Thus, the nematode EF Ts  [ **TSF** ]  appears to have a broad specificity for EF Tu  [ **TUFA** ]  molecules from different species.  Ref: 12490713 Nucleic Acids Res, 2002
- The kinetic results suggest that the initial contact of EF Ts  [ **TSF** ]  with helix D of EF Tu  [ **TUFA** ]  weakens binding interactions around the guanine base.  Ref: 11744709 J Biol Chem, 2002
- Rate constants of EF Ts  [ **TSF** ]  binding to EF Tu  [ **TUFA** ]  in the free or nucleotide bound form or of GDP GTP binding to the EF Tu  [ **TUFA** ] .EF Ts  [ **TSF** ]  complex range from 0.6 x 10 7 to 6 x 10 7 M 1 s 1.  Ref: 11772013 Biochemistry, 2002
- Dissociation of EF Ts  [ **TSF** ]  from the ternary complexes with EF Tu  [ **TUFA** ]  and GDP GTP is 10 3 10 4 times faster than from the binary complex EF Tu  [ **TUFA** ] .EF Ts  [ **TSF** ] , indicating different structures and or interactions of the factors in the binary and ternary complexes.  Ref: 11772013 Biochemistry, 2002
- Recombinant nematode EF Ts  [ **TSF** ]  purified from Escherichia coli bound both of these molecules and also stimulated the translational activity of EF Tu  [ **TUFA** ] , indicating that the nematode EF Ts  [ **TSF** ]  homolog is a functional EF Ts  [ **TSF** ]  protein of mitochondria.  Ref: 12490713 Nucleic Acids Res, 2002
- EF Ts  [ **TSF** ]  enhances the dissociation of GDP and GTP from EF Tu  [ **TUFA** ]  by factors of 6 x 10 4 and 3 x 10 3, respectively.  Ref: 11772013 Biochemistry, 2002

**Non-interactive relationship** (e.g. studied together, co-existance, homology, etc.)

- Previous studies have shown that when bovine mitochondrial elongation factor Ts  [ **TSF** ]  EF Ts  [ **TSF** ]  is expressed in Escherichia coli, it forms a tightly associated complex with E. coli elongation factor Tu  [ **TUFA** ]  EF Tu  [ **TUFA** ] .  Ref: 12445479 Biochim Biophys Acta, 2002
- Mechanism of elongation factor EF Ts  [ **TSF** ]  catalyzed nucleotide exchange in EF Tu  [ **TUFA** ] .  Ref: 11744709 J Biol Chem, 2002
- The interaction of Escherichia coli elongation factor Tu  [ **TUFA** ]  EF Tu  [ **TUFA** ]  with elongation factor Ts  [ **TSF** ]  EF Ts  [ **TSF** ]  and guanine nucleotides was studied by the stopped flow technique, monitoring the fluorescence of tryptophan 184 in EF Tu  [ **TUFA** ]  or of the mant group attached to the guanine nucleotide.  Ref: 11772013 Biochemistry, 2002
- The rate constants of the multistep reaction between Escherichia coli EF Tu  [ **TUFA** ] , EF Ts  [ **TSF** ] , and GDP were determined by stopped flow kinetic analysis monitoring the fluorescence of either Trp 184 in EF Tu  [ **TUFA** ]  or mant GDP.  Ref: 11744709 J Biol Chem, 2002
- Although the nematode mitochondrial mt EF Tu  [ **TUFA** ]  molecules are extremely unstable and easily form aggregates, native gel electrophoresis and gel filtration analysis revealed that EF Tu  [ **TUFA** ] .EF Ts  [ **TSF** ]  complexes are significantly more soluble.  Ref: 12490713 Nucleic Acids Res, 2002
- Rate constants of all association and dissociation reactions among EF Tu  [ **TUFA** ] , EF Ts  [ **TSF** ] , GDP, and GTP were determined.  Ref: 11772013 Biochemistry, 2002
- The crystals of the homologous mitochondrial EF Tu  [ **TUFA** ]  EF Ts  [ **TSF** ]  complex diffract to 4 A and belong to space group C2 with cell parameters a=157.7 A, b=151.9 A, c=156.9 A, and beta=108.96 degrees.  Ref: 12445479 Biochim Biophys Acta, 2002
- We prepared antibodies against EF Tu  [ **TUFA** ]  mt and EF Ts  [ **TSF** ]  mt using purified recombinant EF Tu  [ **TUFA** ]  mt and EF Ts  [ **TSF** ]  mt.  Ref: 12142912 Sheng Wu Hua Xue Yu Sheng Wu Wu Li Xue Bao Shanghai < MedlineTA>Sheng Wu Hua Xue Yu Sheng Wu Wu Li Xue Bao Shanghai , 2002
- In the present paper, we determine the contribution of the contacts between helix D of EF Tu  [ **TUFA** ]  at the base side of the nucleotide and the N terminal domain of EF Ts  [ **TSF** ]  to the catalysis.  Ref: 11744709 J Biol Chem, 2002
- Isolation, crystallisation, and preliminary X ray analysis of the bovine mitochondrial EF Tu  [ **TUFA** ]  GDP and EF Tu  [ **TUFA** ]  EF Ts  [ **TSF** ]  complexes.  Ref: 12445479 Biochim Biophys Acta, 2002
- The loss of Mg 2 alone, without EF Ts  [ **TSF** ] , accounts for a 150 300 fold acceleration of GDP dissociation from EF Tu  [ **TUFA** ] .GDP, suggesting that the disruption of the Mg 2 binding site alone does not explain the EF Ts  [ **TSF** ]  effect.  Ref: 11772013 Biochemistry, 2002
- Kinetic mechanism of elongation factor Ts  [ **TSF** ]  catalyzed nucleotide exchange in elongation factor Tu  [ **TUFA** ] .  Ref: 11772013 Biochemistry, 2002
- The mitochondrial elongation factors Tu and Ts EF Tu  [ **TUFA** ]  mt and EF Ts  [ **TSF** ]  mt are nuclear gene encoded mitochondrial proteins and play important roles in growth and development.  Ref: 12142912 Sheng Wu Hua Xue Yu Sheng Wu Wu Li Xue Bao Shanghai < MedlineTA>Sheng Wu Hua Xue Yu Sheng Wu Wu Li Xue Bao Shanghai , 2002
- Microcalorimetric study of elongation factor Tu  [ **TUFA** ]  from Thermus thermophilus in nucleotide free, GDP and GTP forms and in the presence of elongation factor Ts  [ **TSF** ] .  Ref: 12007615 Biochim Biophys Acta, 2002
- Several proteins, including the elongation factors EF G,  **TufA**  and EF Ts  [ **TSF** ] , were found to be highly carbonylated.  Ref: 12845527 Mol Genet Genomics< MedlineTA>Mol Genet Genomics, 2002
- The bovine mitochondrial EF Tu  [ **TUFA** ]  GDP complex, the homologous mitochondrial EF Tu  [ **TUFA** ]  EF Ts  [ **TSF** ]  complex, and the heterologous E. coli mitochondrial EF Tu  [ **TUFA** ]  EF Ts  [ **TSF** ]  complex were isolated and crystallised.  Ref: 12445479 Biochim Biophys Acta, 2002
- Mutational analysis shows that contacts between helix D of EF Tu  [ **TUFA** ]  and the N terminal domain of EF Ts  [ **TSF** ]  are important for both complex formation and the acceleration of GDP dissociation.  Ref: 11744709 J Biol Chem, 2002
- These results suggest that expression levels of EF Tu  [ **TUFA** ]  mt and EF Ts  [ **TSF** ]  mt may be regulated by a similar mechanism related to the metabolic state of the cells.  Ref: 12142912 Sheng Wu Hua Xue Yu Sheng Wu Wu Li Xue Bao Shanghai < MedlineTA>Sheng Wu Hua Xue Yu Sheng Wu Wu Li Xue Bao Shanghai , 2002
- Expression of EF Tu  [ **TUFA** ]  mt and EF Ts  [ **TSF** ]  mt exhi bited similar tissue specificity and changed synchronically during development.  Ref: 12142912 Sheng Wu Hua Xue Yu Sheng Wu Wu Li Xue Bao Shanghai < MedlineTA>Sheng Wu Hua Xue Yu Sheng Wu Wu Li Xue Bao Shanghai , 2002
- Western analysis indicates EF Tu  [ **TUFA** ]  mt and EF Ts  [ **TSF** ]  mt are expressed differently in various tissues.  Ref: 12142912 Sheng Wu Hua Xue Yu Sheng Wu Wu Li Xue Bao Shanghai < MedlineTA>Sheng Wu Hua Xue Yu Sheng Wu Wu Li Xue Bao Shanghai , 2002
- Although the EF Ts  [ **TSF** ]  did not bind to bovine mt EF Tu  [ **TUFA** ] , it could bind to a chimeric nematode bovine EF Tu  [ **TUFA** ]  molecule containing domains 1 and 2 from bovine mt EF Tu  [ **TUFA** ] .  Ref: 12490713 Nucleic Acids Res, 2002
